# Nutritional supplementation remodels yak cardiac molecular networks through selenoprotein activation and immune quiescence

**DOI:** 10.3389/fnut.2026.1770178

**Published:** 2026-04-14

**Authors:** Yiqun Chen, Chaoyun Yang, Tao Li, Guojun Chen, Yicheng Wang, Zhiqiang Hu, Jun Li, Jing Wang, Ran Guan, Zengwen Huang

**Affiliations:** College of Animal Science, Xichang University, Xichang, China

**Keywords:** Muli yak, multi-omics integration, myocardial adaptation, nutritional stress, post-transcriptional regulation, selenoproteome

## Abstract

Seasonal nutritional stress during prolonged winters represents a pivotal yet understudied environmental determinant constraining livestock productivity on the Qinghai-Tibet Plateau. While cardiac adaptive mechanisms to chronic hypoxia have been extensively characterized in high-altitude ungulates, the molecular architecture underlying myocardial responses to nutritional perturbation remains largely unexplored. Here, we present the first integrative transcriptomic-proteomic atlas of yak cardiac tissue under differential nutritional regimens. Twelve male *Muli yaks* were allocated to ad libitum grazing (GRA, *n* = 18) and winter supplementary feeding (SUP, *n* = 18) cohorts using a completely randomized design over a 60-day intervention period. Myocardial specimens were interrogated via RNA sequencing and data-independent acquisition mass spectrometry. Differential expression analysis yielded 140 DEGs (48 upregulated, 92 downregulated) and 254 DEPs (79 upregulated, 175 downregulated). Nine-quadrant integration across 4,080 cognate gene-protein pairs unveiled a striking decoupling between transcriptional and translational landscapes: merely 3.4% exhibited concordant directionality, whereas 87.3% manifested protein-exclusive alterations, implicating post-transcriptional governance as the predominant regulatory paradigm. The selenoproteome emerged as a convergent nodal point, with *GPX1* (mRNA/protein log₂FC: +0.73/+1.89) and *SELENOW* (+2.11/+3.00) demonstrating coordinated bi-level induction. Reciprocally, stress-responsive immunosurveillance effectors including ULBP13 (mRNA log₂FC = −10.24) and *GZMB* (protein log₂FC = −1.50) underwent pronounced attenuation. *GPX1* and *SELENOW* exhibited flawless discriminatory capacity (AUC = 1.00) across both molecular strata. Collectively, these findings delineate a previously unrecognized regulatory axis whereby nutritional rehabilitation orchestrates myocardial molecular homeostasis through selenoprotein-mediated redox fortification coupled with immunoquiescent reprogramming, positioning *GPX1* and *SELENOW* as robust sentinel biomarkers for nutritional status surveillance in high-altitude ruminants.

## 1 Introduction

The Qinghai-Tibet Plateau, with an average elevation exceeding 4,000 meters, is characterized by an atmospheric oxygen partial pressure of only 60–70% of that at sea level, rendering it one of the most representative extreme hypoxic environments globally (1, 2). Prolonged exposure to hypoxia has been shown to induce pulmonary vasoconstriction and pulmonary arterial hypertension, which subsequently results in a sustained elevation of right ventricular afterload, thereby imposing formidable challenges on the structural and functional integrity of the cardiovascular system (3, 4). The heart, as one of the most energy-demanding organs, is highly dependent on adequate oxygen supply and continuous metabolic substrate availability for the maintenance of normal pumping function (5). Consequently, elucidating the molecular regulatory mechanisms by which the heart sustains functional homeostasis under high-altitude conditions is of considerable scientific significance for understanding the physiological basis of adaptation to extreme environments. Genomic studies have revealed significant positive selection signatures in genes associated with hypoxia sensing, including EPAS1 and ADAM17 (6). Recent whole-genome resequencing studies have further identified copy number variations associated with hypoxia adaptation across diverse yak populations, providing additional genetic evidence for the unique adaptive capacity of this species (7), while morphological investigations have demonstrated that the yak heart is characterized by a relatively larger heart-to-body weight ratio and an extensive capillary network (8). Nevertheless, these studies have predominantly focused on the intrinsic adaptive traits at the genetic level, and a systematic understanding of how environmental factors—particularly nutritional status—dynamically regulate the molecular expression profile of the heart remains lacking.

Indeed, nutritional insufficiency represents a critical environmental factor constraining the physiological performance of high-altitude livestock. The prolonged winter season on the Qinghai-Tibet Plateau is marked by pasture senescence, during which crude protein content can decline to as low as 3–6%, far below the maintenance requirements of yaks (9), resulting in body weight losses of 20–30% (10). For the heart, such nutritional stress may exacerbate the adverse effects of hypoxic challenge through multiple mechanisms: insufficient supply of energy substrates (glucose and fatty acids) directly limits the efficiency of myocardial ATP synthesis (11); deficiency of essential trace elements such as selenium may suppress the expression of antioxidant selenoproteins including glutathione peroxidases, thereby compromising the cardiac defense capacity against oxidative damage (12); furthermore, negative protein balance may impair the turnover and synthesis of contractile proteins (13). In light of these considerations, winter dietary supplementation has been widely adopted as an effective strategy to ameliorate the nutritional status of yaks (14); however, the underlying mechanisms by which such intervention influences cardiac molecular regulatory networks have yet to be comprehensively elucidated. Defining how dietary supplementation remodels myocardial metabolic and defense systems at both transcriptional and translational levels constitutes a key scientific question for uncovering the interplay among nutrition, gene expression, and environmental adaptation.

Against this background, the present study employed RNA sequencing in conjunction with data-independent acquisition proteomics to systematically compare cardiac molecular profiles between traditional grazing and winter supplementary feeding regimens in Muli yaks. The objectives were to: (1) identify differentially expressed genes and proteins responsive to nutritional intervention; (2) delineate the core regulatory networks modulating cardiac adaptation; and (3) screen candidate biomarkers with concordant transcriptional-translational changes for nutritional status assessment in plateau livestock.

## 2 Materials and methods

### 2.1 Experimental design and ethics statement

The experiment was conducted at Yazu Ranch (elevation: 3,793 m) in Muli County, Liangshan Prefecture, Sichuan Province, China. Thirty-six healthy male *Muli yaks* (age: 1.5 ± 0.3 years; body weight: 142 ± 8 kg) were randomly allocated into two groups: the grazing group (GRA, *n* = 18) and the supplementary feeding group (SUP, *n* = 18). Animals in both groups were managed under a rotational grazing system on natural winter pasture with *ad libitum* access to water. *Yaks* in the GRA group were fed exclusively on native pasture (crude protein: 5.2% on a dry matter basis), whereas those in the SUP group received daily concentrate supplementation (1.2% of body weight) in addition to grazing. The concentrate was composed of corn (44%), wheat bran (20%), soybean meal (16%), oats (15%), and mineral–vitamin premix (5%), with a crude protein content of 11.5% (dry matter basis). The experimental period lasted 60 days (From 1 November 2024 to 30 December 2025).

Upon completion of the trial, six animals per group were randomly selected and euthanized by exsanguination following captive bolt stunning. Cardiac tissues (left ventricular myocardium, approximately 500 mg per sample) were excised within 15 min of death, immediately snap-frozen in liquid nitrogen, and stored at −80 °C until trail. All experimental procedures were approved by the Animal Ethics Committee of Xichang University (Approval No.: xcc2025003) and were conducted in accordance with the Guidelines for the Care and Use of Laboratory Animals of China.

### 2.2 Transcriptomic analysis

#### 2.2.1 RNA extraction and quality assessment

Total RNA was extracted from approximately 100 mg of cardiac tissue using TRIzol® Reagent (Invitrogen, Carlsbad, CA, USA) according to the manufacturer’s protocol. RNA concentration was measured using a NanoDrop 2000 spectrophotometer (Thermo Fisher Scientific, Waltham, MA, USA). RNA integrity was assessed using an Agilent 2,100 Bioanalyzer with the RNA 6000 Nano Kit (Agilent Technologies, Santa Clara, CA, USA). Only samples with an RNA Integrity Number (RIN) > 7.0 and A260/A280 ratio between 1.8 and 2.1 were used for library construction.

#### 2.2.2 Library construction and sequencing

Sequencing libraries were prepared from 1 μg of total RNA per sample using the NEBNext® Ultra™ RNA Library Prep Kit for Illumina® (New England Biolabs, Ipswich, MA, USA) following the manufacturer’s instructions. Briefly, poly(A) mRNA was enriched using oligo(dT)-conjugated magnetic beads, fragmented at 94 °C for 15 min, and reverse-transcribed into first-strand cDNA using random hexamer primers. Second-strand cDNA synthesis was performed, followed by end repair, dA-tailing, and adaptor ligation. The ligated fragments were size-selected (insert size: 150–200 bp) using AMPure XP beads (Beckman Coulter, Brea, CA, USA) and amplified by 12 cycles of PCR. Library quality was assessed using the Agilent 2100 Bioanalyzer with the High Sensitivity DNA Kit (Agilent Technologies), and library concentration was quantified by quantitative PCR (qPCR). The qualified libraries were pooled and sequenced on an Illumina NovaSeq 6000 platform (Illumina, San Diego, CA, USA) using a paired-end 150 bp (PE150) sequencing strategy, generating approximately 8G raw reads per sample.

#### 2.2.3 Quality control and read processing

Raw sequencing reads in FASTQ format were processed using Trimmomatic (v0.39) (15) with the following parameters: ILLUMINACLIP:TruSeq3-PE-2.fa:2:30:10, LEADING:3, TRAILING:3, SLIDINGWINDOW:4:15, and MINLEN:36. Adapter sequences and low-quality bases (Phred quality score < 15) were removed. Read quality before and after trimming was assessed using FastQC (v0.11.9; Babraham Bioinformatics, Cambridge, UK).

#### 2.2.4 Read alignment and gene quantification

Clean reads were aligned to the *Bos taurus* reference genome (assembly ARS-UCD1.3; NCBI accession: GCF_002263795.2) using HISAT2 (v2.2.1) (16) with default parameters. The *Bos taurus* genome was selected as the reference due to the high genomic synteny between yak (*Bos grunniens*) and cattle, and the limited annotation resources available for the yak genome. Alignment files in BAM format were generated and sorted using SAMtools (v1.15) (17). Gene-level expression was quantified using StringTie (v2.2.1) (18) with NCBI RefSeq GTF annotation (release 110). Expression values were normalized to Fragments Per Kilobase of transcript per Million mapped reads (FPKM).

#### 2.2.5 Differential gene expression analysis

Differential expression analysis was performed using the DESeq2 package (v1.38.0) (19) in R (v4.2.0; R Foundation for Statistical Computing, Vienna, Austria). Raw read counts generated by StringTie were used as input. Genes with fewer than 10 counts across all samples were filtered out. Ambiguously annotated transcripts (those with “LOC” or “Novel” prefixes) were excluded from downstream analysis. Gene symbol annotation was performed using the org.Bt.eg.db database (v3.16.0; Bioconductor). For genes mapping to multiple transcripts, the transcript with the highest mean expression was retained. Differentially expressed genes (DEGs) between the SUP (*n* = 6) and GRA (*n* = 6) groups were identified using thresholds of Benjamini–Hochberg (BH) adjusted *p*-value <0.05 and |log_2_fold change (FC)| > 0.585 (corresponding to FC > 1.5).

#### 2.2.6 Functional enrichment analysis of DEGs

Gene symbols of DEGs were converted to Entrez Gene IDs using the *bitr()* function from the clusterProfiler package (v4.8.0) (20) with reference to the org.Bt.eg.db annotation database (v3.17.0). Gene Ontology (GO) enrichment analysis for Biological Process (BP) terms was performed using the *enrichGO()* function with the following parameters: *OrgDb = org.Bt.eg.db*, *ont = “ALL,” pAdjustMethod = “BH,” pvalueCutoff = 0.05*, qvalueCutoff = 0.05, and *readable = TRUE.* KEGG pathway enrichment analysis was conducted using the *enrichKEGG()* function with organism code “bta” (*Bos taurus*), *pvalueCutoff = 0.05*, and *qvalueCutoff = 0.05*. Gene IDs were converted to readable gene symbols using the *setReadable()* function.

Core genes were selected using a two-tier strategy: (i) hub genes participating in ≥2 enriched pathways were prioritized; (ii) if the number of hub genes was insufficient (<20 genes), supplementation with high-impact genes (top 3 by |log₂FC| per pathway) was performed. The top 15 GO-BP terms and top 10 KEGG pathways (ranked by adjusted *p*-value) were selected for visualization.

Heatmaps were generated using the ComplexHeatmap package (v2.16.0) (21) with the following specifications: a blue–white–red color gradient centered at zero was created using the *colorRamp2()* function from the circlize package (v0.4.15), with scale limits determined by the maximum absolute fold change; left annotation bars indicated database source (GO: green; KEGG: orange); right annotation bar plots displayed −log₁₀(adjusted *p*-value); row and column clustering were disabled; pathways were ordered by adjusted *p*-value within each database category; missing values (NA) were displayed in gray.

### 2.3 Proteomic analysis

#### 2.3.1 Protein extraction and digestion

Frozen cardiac tissue samples (approximately 50 mg each) were homogenized in SDT lysis buffer (4% SDS, 100 mM Tris–HCl pH 7.6, 0.1 M DTT) using a tissue homogenizer (TissueLyser II; Qiagen, Hilden, Germany) at 25 Hz for 2 × 3 min with intermittent cooling on ice. Lysates were heated at 95 °C for 5 min, sonicated on ice (10 cycles: 10 s on, 10 s off), and centrifuged at 14,000 × *g* for 15 min at 4 °C. The supernatant was collected, and protein concentration was determined using a BCA Protein Assay Kit (Thermo Fisher Scientific) according to the manufacturer’s instructions.

Protein digestion was performed using the filter-aided sample preparation (FASP) method (22). Briefly, 200 μg of protein per sample was loaded onto a 10 kDa molecular weight cutoff ultrafiltration device (Millipore, Burlington, MA, USA), washed with UA buffer (8 M urea in 0.1 M Tris–HCl, pH 8.5), alkylated with 50 mM iodoacetamide in UA buffer for 30 min in the dark at room temperature, and washed three times with 50 mM NH₄HCO₃. Proteins were digested with sequencing-grade modified trypsin (Promega, Madison, WI, USA; Cat. No. V5113) at an enzyme-to-protein ratio of 1:50 (w/w) at 37 °C for 16 h. Peptides were eluted by centrifugation, desalted using C18 StageTips, and dried under vacuum.

#### 2.3.2 LC–MS/MS analysis

Peptide samples were reconstituted in 0.1% formic acid (FA) and analyzed using a nanoElute liquid chromatography system (Bruker Daltonics, Bremen, Germany) coupled to a timsTOF Pro mass spectrometer (Bruker Daltonics). Peptides (approximately 200 ng per injection) were loaded onto a C18 trap column (100 μm × 2 cm, 3 μm particle size) and separated on an analytical column (75 μm × 25 cm, 1.9 μm particle size; IonOpticks, Melbourne, Australia) at a flow rate of 300 nL/min using a 90 min linear gradient of 2–35% mobile phase B (0.1% FA in acetonitrile) in mobile phase A (0.1% FA in water).

Mass spectrometric analysis was performed in data-independent acquisition (DIA) mode. Full MS scans were acquired over the *m/z* range of 100–1700 with a resolution of 20,000. DIA windows were set to 25 Da across the *m/z* range of 400–1,200. The ion mobility range was set to 0.60–1.60 *Vs*/cm^2^, and the accumulation and ramp times were both set to 100 ms.

#### 2.3.3 Proteomic data processing

Raw MS data files (.d format) were processed using DIA-NN software (v1.8.1) (23) for spectral library generation and database searching against the *Bos taurus* UniProt reference proteome (UP000009136). Search parameters were set as follows: precursor *m/z* range: 300–1800; fragment ion *m/z* range: 200–1800; precursor charge: 2–4; missed cleavages: 2; fixed modification: carbamidomethylation of cysteine (+57.021 Da); variable modification: oxidation of methionine (+15.995 Da). The false discovery rate (FDR) was controlled at < 1% at both peptide and protein levels using the target-decoy approach (*q*-value < 0.01). Protein quantification was performed using the MaxLFQ algorithm implemented in DIA-NN with match-between-runs enabled.

#### 2.3.4 Differential protein expression analysis

Statistical analyses were conducted using R (v4.3.3). Proteins with “LOC” prefixes (unannotated) and those with >50% missing values across samples were excluded. Missing values in the remaining proteins were imputed using the k-nearest neighbors (kNN) algorithm with *k* = 5. Protein abundance values were log_2_-transformed and median-normalized across samples. Differentially expressed proteins (DEPs) between SUP and GRA groups were identified using a two-tailed Student’s *t*-test with Benjamini–Hochberg correction for multiple testing. Significance thresholds were set at adjusted *p*-value < 0.05 and |log_2_FC| > 0.263 (corresponding to FC > 1.2). The less stringent fold-change threshold for proteomic analysis was applied to account for the inherently lower dynamic range and higher technical variability in protein quantification compared to RNA sequencing.

### 2.4 Functional enrichment analysis of DEPs

Gene symbols corresponding to DEPs were converted to Entrez Gene IDs using the *bitr()* function from the clusterProfiler package (v4.8.3) with reference to the org.Bt.eg.db annotation database (v3.17.0). GO enrichment analysis was conducted using the *enrichGO()* function with the following parameters: *ont = “ALL”* (including BP, Cellular Component (CC), and Molecular Function (MF) terms), *pAdjustMethod = “BH,” pvalueCutoff = 0.05*, and *qvalueCutoff = 0.1*. KEGG pathway enrichment was performed using the *enrichKEGG()* function with organism code “*bta*,” applying identical *p*-value and *q*-value thresholds. Enrichment results were visualized using dot plots and bar plots generated with the ggplot2 package (v3.4.4).

### 2.5 Multi-omics integration analysis

#### 2.5.1 Data preprocessing and integration

For integrative analysis, genes and proteins were matched based on gene symbol identifiers. The transcriptomic dataset initially comprised 24,900 genes, and the proteomic dataset comprised 4,188 proteins. The following quality control procedures were applied: (i) genes with “LOC” prefixes were excluded from the transcriptomic dataset; (ii) entries with missing or infinite values were removed. An inner join was performed based on gene identifiers to generate matched gene–protein pairs for downstream analysis.

#### 2.5.2 Nine-quadrant classification of regulatory patterns

Matched gene–protein pairs were classified into nine quadrants based on their differential expression status at the transcriptional and translational levels (Table 1). Classification criteria were as follows:

**Table 1:** Nine-quadrant classification of regulatory patterns.

Quadrant	mRNA status	Protein status	Regulatory pattern
Q1	Down	Up	Discordant (post-transcriptional up-regulation)
Q2	NS	Up	Protein-only up-regulation
Q3	Up	Up	Concordant up-regulation
Q4	Down	NS	mRNA-only down-regulation
Q5	NS	NS	No significant change
Q6	Up	NS	mRNA-only up-regulation
Q7	Down	Down	Concordant down-regulation
Q8	NS	Down	Protein-only down-regulation
Q9	Up	Down	Discordant (post-transcriptional down-regulation)

NS (not significant), the significance thresholds for mRNA were |NS (not significant), the significance thresholds for mRNA were |log2FC| > 0.585 and adjusted p < 0.05; for protein, |log2FC| > 0.263 and p < 0.05.|NS (not significant), the significance thresholds for mRNA were |log2FC| > 0.585 and adjusted p < 0.05; for protein, |log2FC| > 0.263 and p < 0.05.|NS (not significant), the significance thresholds for mRNA were |log2FC| > 0.585 and adjusted p < 0.05; for protein, |log2FC| > 0.263 and p < 0.05.|NS (not significant), the significance thresholds for mRNA were |log2FC| > 0.585 and adjusted p < 0.05; for protein, |log2FC| > 0.263 and p < 0.05.

#### 2.5.3 Quadrant-based functional enrichment analysis

Functional enrichment analysis was performed separately for genes within each quadrant. GO-BP and KEGG pathway enrichment analyses were conducted using clusterProfiler (v4.8.0) with *p*-value and *q*-value thresholds of 0.05. The background gene set consisted of all 4,080 matched gene–protein pairs.

#### 2.5.4 Data visualization

Nine-quadrant scatter plots were generated using the ggplot2 package (v3.5.2), with mRNA log_2_FC plotted on the x-axis and protein log_2_FC on the y-axis. Quadrant boundaries were delineated by dashed reference lines at the respective differential expression thresholds. A hierarchical labeling strategy was employed: (i) all genes in Q3 and Q7 (concordant changes) were labeled with gold diamond symbols; (ii) the top 3 genes with the largest magnitude of change in each remaining quadrant (Q1, Q2, Q4, Q6, Q8, Q9) were labeled with red circle symbols. Selection criteria were as follows: genes in Q1, Q4, Q6, Q7, and Q9 were ranked by absolute mRNA log_2_FC; genes in Q2 and Q8 were ranked by absolute protein log₂FC. Venn diagrams illustrating the overlap between DEGs and DEPs were generated using the VennDiagram package (v1.7.3).

### 2.6 Cross-omics correlation and diagnostic performance analysis of Core candidate genes

#### 2.6.1 Core candidate genes were prioritized from the integrative transcriptome-proteome analysis based on the following criteria

(1) Localization in biologically significant quadrants (Q2, Q3, Q7, Q8) of the nine-quadrant plot; (2) involvement in key biological processes identified through functional enrichment; and (3) documented functional relevance in cardiac physiology or stress response. Genes meeting these criteria were subjected to downstream validation analyses.

#### 2.6.2 Cross-omics correlation analysis

For genes quantified at both transcriptome and proteome levels, Pearson correlation coefficients with 95% confidence intervals (CI) were calculated to assess mRNA-protein expression concordance across all samples (*n* = 12). Analyses were conducted using the cor.test() function in R (v4.3.1).

#### 2.6.3 ROC analysis

Receiver operating characteristic curves were constructed to evaluate the discriminatory capacity of each candidate gene between SUP and GRA groups. Area under the curve (AUC) with 95% CI was computed using the pROC package (v1.18.5), with CI estimated via the DeLong method. Diagnostic performance was categorized as excellent (AUC 0.9–1.0), good (AUC 0.8–0.9), acceptable (AUC 0.7–0.8), or poor (AUC < 0.7). All statistical tests were two-sided (*α* = 0.05).

### 2.7 Statistical analysis

All statistical analyses were performed in R (v4.3.0) unless otherwise specified. Continuous variables were expressed as mean ± standard deviation (SD). Between-group comparisons were performed using two-tailed Student’s *t*-tests for normally distributed data or Wilcoxon rank-sum tests for non-normally distributed data. Multiple testing correction was performed using the Benjamini–Hochberg method, with adjusted *p* < 0.05 considered statistically significant. Sample sizes (*n* = 6 per group) were determined based on previous transcriptomic studies in ruminants and practical constraints of animal experimentation.

## 3 Results

### 3.1 Transcriptome sequencing data quality assessment

Key quality metrics from six samples per group (GRA and SUP) were analyzed (Table 2; Supplementary Table S1; Supplementary Figure S1). The GRA and SUP groups generated 56.97 ± 1.16 and 55.96 ± 1.40 million raw reads, respectively, yielding 56.78 ± 1.16 and 55.81 ± 1.41 million clean reads after quality filtering, corresponding to 8.52 ± 0.17 Gb and 8.37 ± 0.20 Gb of high-quality bases. The sequencing error rate was consistently 0.01% across all samples. Q20 scores exceeded 99.4% (GRA: 99.41 ± 0.02%; SUP: 99.43 ± 0.02%), and Q30 scores exceeded 97.4% (GRA: 97.49 ± 0.08%; SUP: 97.56 ± 0.06%). GC content was stable between groups (GRA: 49.70 ± 0.31%; SUP: 49.15 ± 0.66%). Clean read retention rates exceeded 99.6% in both groups (GRA: 99.67 ± 0.01%; SUP: 99.69 ± 0.02%). Two-tailed Student’s *t*-tests revealed no significant differences in any quality metrics between groups (*p* > 0.05 for all comparisons; Table 2). High correlation was observed between SUP and GRA samples (Supplementary Figure S2).

**Table 2:** RNA-seq quality metrics.

Parameter	GRA (*n* = 6)	SUP (*n* = 6)	*p*-value
Raw reads (×10^6^)	56.97 ± 1.16	55.96 ± 1.40	0.186
Clean reads (×10^6^)	56.78 ± 1.16	55.81 ± 1.41	0.203
Raw bases (G)	8.55 ± 0.17	8.40 ± 0.20	0.175
Clean bases (G)	8.52 ± 0.17	8.37 ± 0.20	0.185
Error rate (%)	0.01 ± 0.00	0.01 ± 0.00	1
Q20 (%)	99.41 ± 0.02	99.43 ± 0.02	0.147
Q30 (%)	97.49 ± 0.08	97.56 ± 0.06	0.102
GC content (%)	49.70 ± 0.31	49.15 ± 0.66	0.089
Clean reads ratio (%)	99.67 ± 0.01	99.69 ± 0.02	0.052

Values are presented as mean ± SD.

### 3.2 Identification and characterization of differentially expressed genes

**Figure 1:**
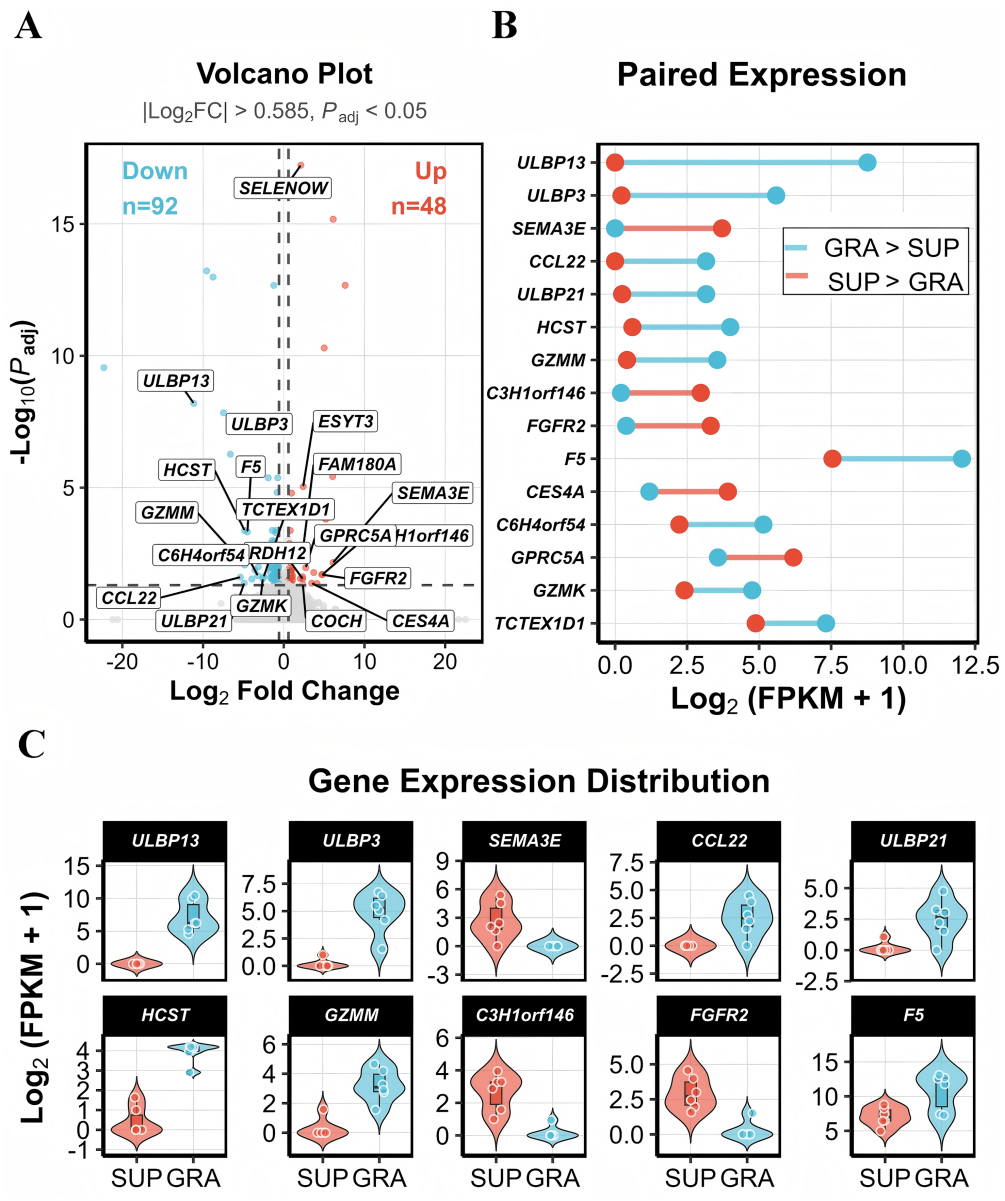
Differential gene expression analysis between SUP and GRA groups. **(A)** Volcano plot of DEGs. Red: upregulated (*n* = 48); blue: downregulated (*n* = 92). Thresholds: |FC| > 1.5, *Padj* < 0.05. **(B)** Paired expression plot of 15 representative DEGs showing mean log_2_(FPKM+1) values. Red lines: SUP > GRA; blue lines: GRA > SUP. **(C)** Violin plots of 10 key DEGs. White dots represent individual replicates (*n* = 6 per group).

Using filtering criteria of |log₂FC| > 0.585 and adjusted *p* < 0.05, a total of 140 differentially expressed genes (DEGs) were identified from 24,901 genes, comprising 48 up-regulated and 92 down-regulated genes in the SUP group relative to the GRA group (Figure 1A; Supplementary Tables S2, S3).

Volcano plot analysis shows the distribution of DEGs (Figure 1A). Among up-regulated genes, *SELENOW* (log₂FC = 2.11), *SEMA3E*, *FAM180A*, *ESYT3*, and *GPRC5A* exhibited the largest fold changes. Among down-regulated genes, members of the *ULBP* family (*ULBP13*, *ULBP3*, *ULBP21*), cytotoxic effectors *GZMM* and *GZMK*, the immune adaptor *HCST*, the chemokine *CCL22*, and coagulation factor F5 showed the largest magnitude of change.

Paired expression analysis of top-ranked DEGs revealed switch-like expression patterns for several genes (Figure 1B). *ULBP13* and *F5* showed high expression in the GRA group (log₂FPKM > 10) but were nearly undetectable in the SUP group (log₂FPKM < 2), representing >100-fold differences. *CCL22* and *ULBP21* displayed similar patterns. Conversely, *SEMA3E* was expressed in the SUP group (log₂FPKM ≈ 4–6) with virtually no expression in GRA samples. *C3H1orf146*, *FGFR2*, and *GPRC5A* showed substantially higher expression in the SUP group. *HCST* and *GZMM* were highly expressed in the GRA group but suppressed in the SUP group.

Violin plots of ten representative DEGs confirmed expression distributions across individual samples (Figure 1C). The *ULBP* gene family (*ULBP13*, *ULBP3*, *ULBP21*) showed high expression in the GRA group (median log_2_FPKM: 7–12) with near-complete silencing in the SUP group. *GZMM* and *HCST* showed stable expression in GRA samples. *SEMA3E*, *GPRC5A*, and *C3H1orf146* were specifically expressed in the SUP group with minimal within-group variation. F5 showed high expression in the GRA group (log₂FPKM ≈ 8–12) with significant reduction in the SUP group. All examined genes demonstrated clear inter-group separation.

The expression profiles showed that the GRA group was characterized by predominant expression of immune-related genes (*ULBP* family, *GZMM*, *GZMK*, *HCST*, *CCL22*), whereas the SUP group exhibited elevated expression of growth factor receptors (*FGFR2*), signaling molecules (*SEMA3E*, *GPRC5A*), and selenoproteins (*SELENOW*).

### 3.3 Functional enrichment analysis of DEGs

Following quality control, 104 of 106 DEGs (98.11%) were successfully mapped to Entrez IDs for enrichment analysis.

#### 3.3.1 GO biological process enrichment

**Figure 2:**
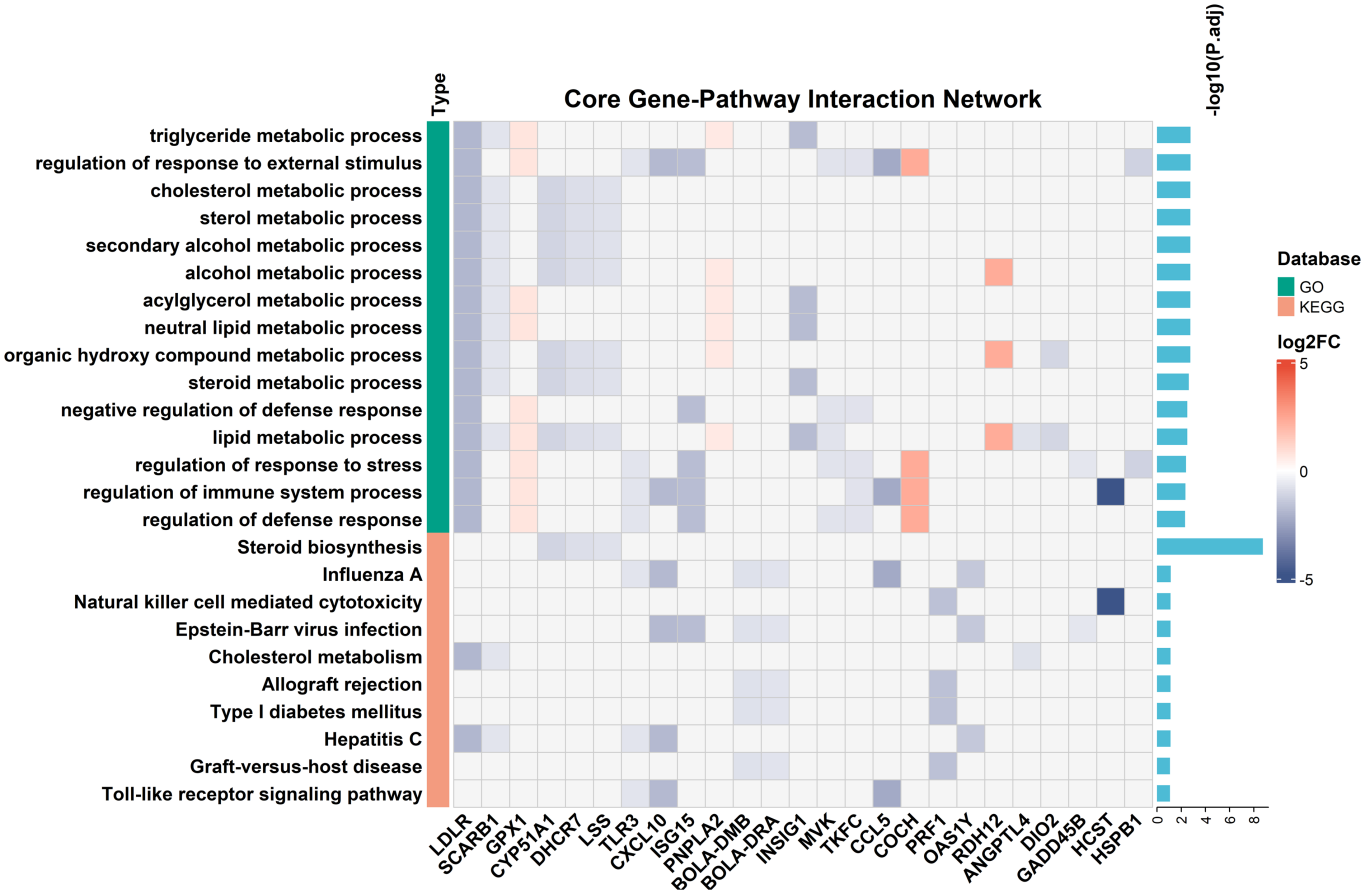
Integrated GO and KEGG enrichment heatmap of cardiac tissue. Heatmap displays log_2_FC values for 25 core genes across 25 enriched pathways (15 GO-BP, 10 KEGG; *Padj* < 0.05). Blue: downregulation; red: upregulation; gray: non-participating. Left bars indicate database source (green: GO; orange: KEGG). Right barplots show -log_10_(*Padj*). LDLR, SCARB1, and GPX1 showed highest pathway connectivity (17, 12, and 9 pathways, respectively).

GO-BP enrichment identified 981 terms (Figure 2; Supplementary Table S4). The top-ranked terms included triglyceride metabolic process (adjusted *p* = 1.71 × 10^−3^; 5 genes), regulation of response to external stimulus (adjusted *p* = 1.71 × 10^−3^; 10 genes), and cholesterol metabolic process (adjusted *p* = 1.76 × 10^−3^; 5 genes). The median adjusted *p*-value across all enriched GO terms was 0.309.

#### 3.3.2 KEGG pathway enrichment

KEGG pathway analysis revealed 148 enriched pathways (adjusted *p* < 0.05; Figure 2; Supplementary Table S4). Steroid biosynthesis showed the strongest enrichment (adjusted *p* = 1.76 × 10^−9^; 7 genes), followed by Influenza A (adjusted *p* = 7.42 × 10^−2^; 6 genes) and Natural killer cell mediated cytotoxicity (adjusted *p* = 7.59 × 10^−2^; 5 genes). The median adjusted *p*-value across KEGG pathways was 0.467.

#### 3.3.3 Identification of core genes

A two-tier gene selection strategy identified 25 core genes from 35 enriched genes (71.43% selection rate), all participating in ≥2 pathways (Figure 2). Five genes showed the highest pathway connectivity: *LDLR* (17 pathways; log₂FC = −1.92), *SCARB1* (12 pathways; log₂FC = −0.71), *GPX1* (9 pathways; log₂FC = +0.73), *CYP51A1* (8 pathways; log₂FC = −1.14), and *DHCR7* (8 pathways; log₂FC = −0.87).

The integrated gene–pathway matrix (25 pathways × 25 genes) achieved 22.4% data density (140/625 non-missing values), with expression fold changes spanning −4.85 to +2.36 (log₂ scale; mean = −0.89). Of the 140 gene–pathway associations, 118 (84.3%) showed down-regulation and 22 (15.7%) showed up-regulation in the SUP group relative to the GRA group (Figure 2). Hierarchical clustering revealed three functional modules:

##### 3.3.3.1 Lipid metabolism module

Encompassing triglyceride metabolic process, cholesterol metabolic process, and sterol metabolic process, with coordinated down-regulation of *LDLR* (log₂FC = −1.92), *CYP51A1* (log₂FC = −1.14), and *DHCR7* (log₂FC = −0.87). The KEGG pathway Steroid biosynthesis (adjusted *p* = 1.76 × 10^−9^) showed uniform down-regulation across all 7 participating genes (Figure 2; Supplementary Table S3).

##### 3.3.3.2 Antioxidant module

*GPX1* (log₂FC = +0.73) was among the few up-regulated genes, participating in 9 pathways.

##### 3.3.3.3 Immune-related module

Natural killer cell mediated cytotoxicity (adjusted *p* = 7.59 × 10^−2^), Toll-like receptor signaling, Influenza A, and Type I diabetes mellitus pathways showed mixed expression patterns with both up- and down-regulated genes. All 25 displayed pathways maintained statistical significance (adjusted *p*: 1.76 × 10^−9^ to 8.42 × 10^−2^; median = 3.10 × 10^−3^). *LDLR* and *SCARB1* participated in 17 and 12 pathways, respectively, representing the most highly connected genes in the network.

### 3.4 Proteomic data quality and identification of DEPs

#### 3.4.1 Data quality assessment

A total of 4,345 proteins and 41,761 unique peptides were identified in *yak* cardiac tissues using DIA-based quantitative proteomics. Principal component analysis (PCA) demonstrated clear separation between the GRA (*n* = 6) and SUP (*n* = 6) groups, with samples clustering by group (Supplementary Figure S4A). Coefficient of variation (CV) analysis showed that the majority of proteins in both groups had CV values below 0.3 (Supplementary Figure S4B).

#### 3.4.2 Identification of differentially expressed proteins

Gene symbol conversion to Entrez IDs achieved 100% success for up-regulated proteins (83/83) and 98.9% for down-regulated proteins (173/175), with only two proteins (SARS and LARS) failing to map. In total, 256 DEPs were successfully annotated for enrichment analysis.

### 3.5 Functional enrichment analysis of DEPs

#### 3.5.1 Enrichment of up-regulated proteins

**Figure 3:**
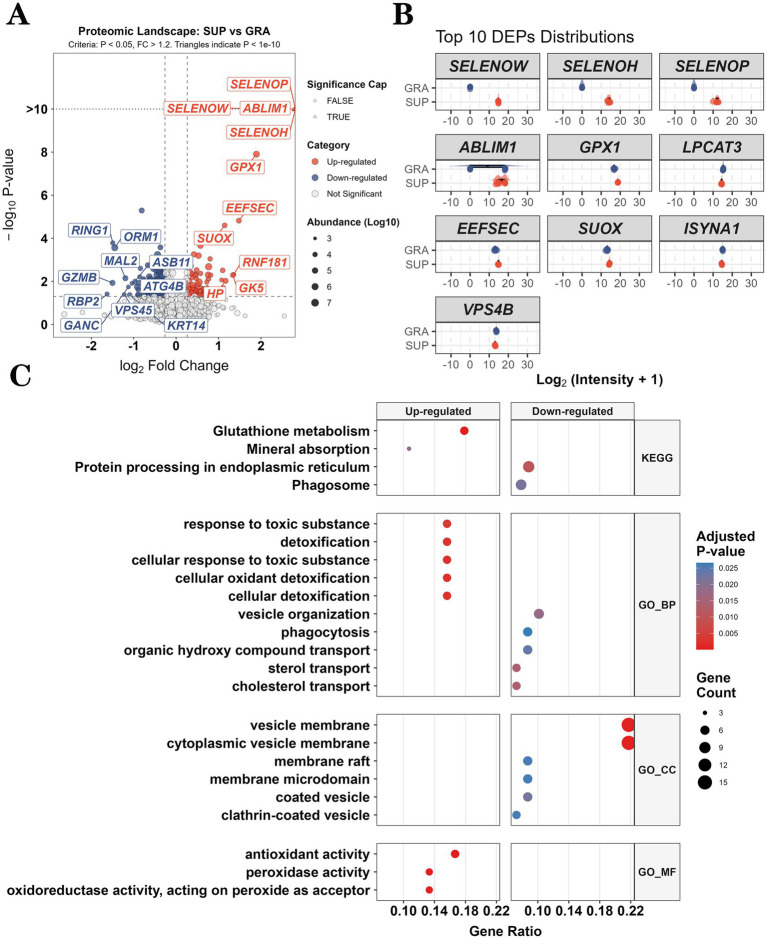
Proteomic profiling and functional enrichment of DEPs in yak cardiac tissue. **(A)** Volcano plot of DEPs (*n* = 258; 83 up, 175 down) from 4,188 quantified proteins. Thresholds: FC > 1.2 or < 0.833, *P* < 0.05. Bubble size indicates mean abundance; triangles denote *P* < 1×10_-10_. Top 10 DEPs per direction are labeled. **(B)** Raincloud plots showing abundance distributions of top 10 DEPs across replicates (*n* = 6 per group). **(C)** GO and KEGG enrichment of DEPs (*Padj* < 0.05). Bubble size: gene count; color: *Padj*. Up-regulated proteins enriched in antioxidant activity; down-regulated proteins enriched in vesicle membrane components.

GO enrichment of the 83 up-regulated proteins revealed 8 significant terms (adjusted *p* < 0.05): 5 Biological Process (BP) and 3 Molecular Function (MF) terms (Figure 3A; Supplementary Table S5). The MF category was dominated by antioxidant-related terms: antioxidant activity (adjusted *p* = 2.33 × 10^−4^), peroxidase activity (adjusted *p* = 3.35 × 10^−4^), and oxidoreductase activity acting on peroxide as acceptor (adjusted *p* = 3.35 × 10^−4^). The BP category included cellular oxidant detoxification (adjusted *p* = 2.85 × 10^−3^) and cellular response to toxic substance (adjusted *p* = 2.85 × 10^−3^).

KEGG pathway analysis identified two significantly enriched pathways: Glutathione metabolism (adjusted *p* = 4.46 × 10^−5^) and Mineral absorption (adjusted *p* = 1.93 × 10^−2^).

#### 3.5.2 Enrichment of down-regulated proteins

GO enrichment of the 173 down-regulated proteins identified 16 significant terms (adjusted *p* < 0.05): 7 BP and 9 Cellular Component (CC) terms (Figure 3B; Supplementary Table S5). The CC category was enriched in membrane-associated structures: cytoplasmic vesicle membrane and vesicle membrane (both adjusted *p* = 6.02 × 10^−4^), endoplasmic reticulum membrane (adjusted *p* = 1.72 × 10^−2^), and organelle membrane (adjusted *p* = 1.92 × 10^−2^). The BP category included cholesterol transport and sterol transport (both adjusted *p* = 1.36 × 10^−2^), vesicle organization (adjusted *p* = 1.82 × 10^−2^), and response to hypoxia (adjusted *p* = 2.58 × 10^−2^).

KEGG analysis revealed two enriched pathways: protein processing in endoplasmic reticulum (adjusted *p* = 1.08 × 10^−2^) and Phagosome (adjusted *p* = 2.18 × 10^−2^).

#### 3.5.3 Identification of hub proteins

Among up-regulated proteins, five genes (*GPX1*, *GPX3*, *GPX4*, *TXNRD1*, and *HP*) appeared in all 8 enriched GO terms (5 BP + 3 MF). Four genes (*GPX1*, *GPX3*, *GPX4*, and *GGCT*) also participated in the Glutathione metabolism KEGG pathway, with *GPX1*, *GPX3*, and *GPX4* forming a core glutathione peroxidase cluster present in both GO and KEGG enrichments.

Among down-regulated proteins, multi-pathway participation was more dispersed. *LAMTOR1*, *ARL8B*, and *VPS4B* each appeared in 5 different terms. *VAMP7* and *RAB5A* were present in 6 terms. The vacuolar ATPase subunits *ATP6V0D1* and *ATP6V0A1* appeared in 9 CC terms and both KEGG pathways. *TMED10* participated in 7 terms across BP, CC, and KEGG categories.

#### 3.5.4 Comparison of enrichment profiles

The enrichment profiles of up- and down-regulated proteins showed distinct functional patterns (Figure 3; Table 3). Up-regulated proteins were enriched in catalytic activities related to antioxidant defense (3 MF terms) and oxidative stress responses (5 BP terms). Down-regulated proteins were enriched in structural components of membrane systems (9 CC terms) and processes related to lipid transport and vesicular trafficking (7 BP terms).

**Table 3:** Summary of functional enrichment analysis.

Parameter	Up-regulated	Down-regulated
DEPs analyzed	83	173
Successfully mapped	83 (100%)	173 (98.9%)
Enriched GO terms	8 (5 BP, 3 MF)	16 (7 BP, 9 CC)
Enriched KEGG pathways	2	2
Top GO term	Antioxidant activity	Vesicle membrane
Top KEGG pathway	Glutathione metabolism	Protein processing in ER

### 3.6 Integrated transcriptomic–proteomic analysis

#### 3.6.1 Overview of differential expression at transcriptomic and proteomic levels

Among 17,251 genes analyzed, 116 DEGs (0.67%) were identified, comprising 41 up-regulated and 75 down-regulated genes. Among 4,183 proteins analyzed, 254 DEPs (6.07%) were identified, comprising 79 up-regulated and 175 down-regulated proteins. The proportion of DEPs was significantly higher than that of DEGs (6.07% vs. 0.67%; *χ*^2^ test, *p* < 0.001). The protein log₂FC range (−1.63 to 1.89) was narrower than the mRNA log₂FC range (−11.16 to 6.11).

#### 3.6.2 Nine-quadrant classification of gene–protein pairs

**Figure 4:**
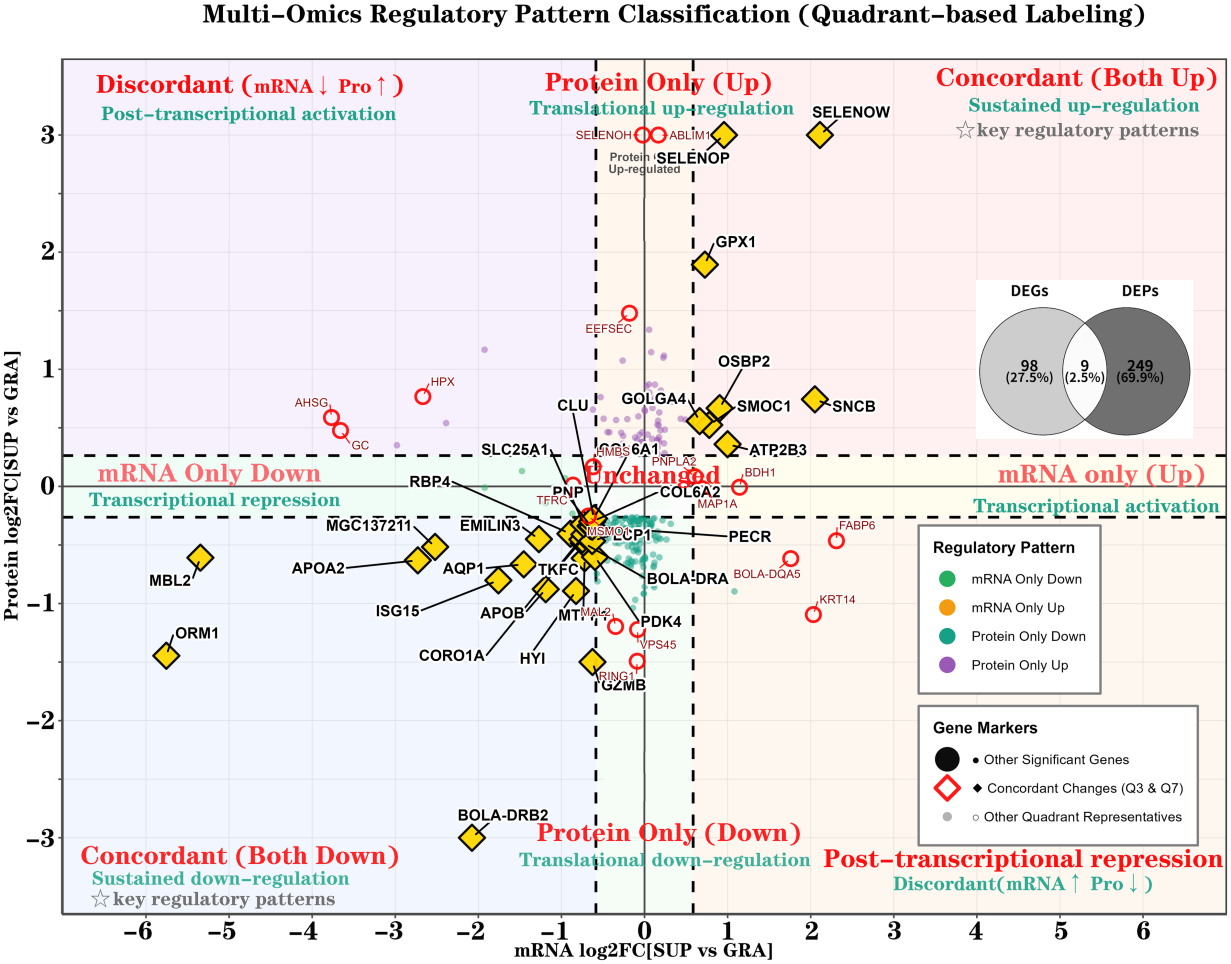
Nine-quadrant classification of transcriptome-proteome regulatory patterns. Scatter plot correlating mRNA and protein log_2_FC for 4,080 matched pairs. Significance thresholds: mRNA (|log_2_FC| > 0.585, *Padj* < 0.05); protein (|log_2_FC| > 0.263, *P* < 0.05). Quadrants: Q3/Q7, concordant up/down; Q1/Q9, discordant; Q4/Q6, mRNA-only; Q2/Q8, protein-only. Gold diamonds: concordant changes; red circles: top representatives from other quadrants..

Among 4,080 matched gene–protein pairs, 268 pairs (6.57%) exhibited significant changes at one or both omics levels, while 3,812 pairs (93.43%) remained unchanged (Figure 4; Table 4; Supplementary Table S6). Only 9 genes (3.4% of changed pairs) showed concordant changes at both transcriptional and translational levels (Q3 and Q7), whereas 234 proteins (87.3%) changed independently of their corresponding mRNA levels (Q2 and Q8).

**Table 4:** Distribution of gene-protein pairs across nine quadrants.

Quadrant	Pattern	Pairs (%)	Top 3 representative genes
Q3	Concordant up	3 (0.07)	*SELENOW*, *SELENOP*, *GPX1*
Q7	Concordant down	6 (0.15)	*AQP1*, *HYI*, *TKFC*
Q1	mRNA↓Protein↑	7 (0.17)	*AHSG*, *GC*, *HPX*
Q9	mRNA↑Protein↓	4 (0.10)	*FABP6*, *KRT14*, *BOLA*-*DQA5*
Q6	mRNA only up	4 (0.10)	*BDH1*, *MAP1A*, *PNPLA2*
Q4	mRNA only down	10 (0.25)	*LDLR*, *TFRC*, *MSMO1*
Q2	Protein only up	69 (1.69)	*ABLIM1*, *SELENOH*, *EEFSEC*
Q8	Protein only down	165 (4.04)	*RING1*, *VPS45*, *MAL2*

##### 3.6.2.1 Concordant regulation (Q3 and Q7)

The three genes in Q3 (SELENOW, SELENOP, GPX1) displayed coordinated up-regulation at both levels, with mRNA log₂FC ranging from 0.73 to 2.11 and protein log₂FC ranging from 1.89 to 3.00. All three genes belong to the selenoprotein family. The six genes in Q7 showed concordant down-regulation: *AQP1* (mRNA/protein log₂FC: −1.45/−0.67), *HYI* (−0.82/−0.89), *TKFC* (−0.75/−0.45), *SLC25A1* (−0.73/−0.32), *BOLA-DRA* (−0.73/−0.48), and *PNP* (−0.65/−0.34).

##### 3.6.2.2 Discordant regulation (Q1 and Q9)

Seven genes in Q1 exhibited mRNA down-regulation but protein up-regulation, with mean mRNA log₂FC of −3.18 and mean protein log₂FC of +0.67. These included *AHSG* (−3.77/+0.59), *GC* (−3.66/+0.48), *HPX* (−2.67/+0.77), *TF* (−2.39/+0.54), and *HP* (−1.93/+1.17). Four genes in Q9 showed the opposite pattern: FABP6 (+2.31/−0.46), KRT14 (+2.03/−1.09), *BOLA-DQA5* (+1.76/−0.62), and *KRT79* (+1.08/−0.90).

##### 3.6.2.3 Single-level regulation (Q2, Q4, Q6, and Q8)

Four genes in Q6 showed mRNA-only up-regulation: *BDH1* (log₂FC = 1.14), *MAP1A* (0.70), *PNPLA2* (0.61), and *ACAD9* (0.60). Ten genes in Q4 displayed mRNA-only down-regulation, including *LDLR* (−1.92), *TFRC* (−0.86), *MSMO1* (−0.68), and *DHCR7* (−0.87). Protein-only changes (Q2 and Q8) accounted for 87.3% of all significantly changed gene–protein pairs (234/268 pairs). Among 69 proteins in Q2, ABLIM1 (log₂FC = 3.00), SELENOH (3.00), and EEFSEC (1.48) exhibited the highest protein log₂FC values. Additional proteins in Q2 included GPX3 (0.75), GPX4 (0.57), TXNRD1 (0.42), and SUOX (1.15). Q8 contained 165 proteins with protein-only down-regulation, including RING1 (−1.49), VPS45 (−1.22), MAL2 (−1.20), GZMB (−1.50), ACSL1 (−0.44), LPCAT3 (−0.81), PDK4 (−0.60), and ATG4B (−1.04).

The nine-quadrant scatter plot (Figure 4) displays the spatial distribution of 50 labeled representative genes. Concordant changes (Q3 and Q7; 9 genes) are marked with gold diamonds; top genes by magnitude in other quadrants are marked with red circles.

### 3.7 Quadrant-based functional enrichment analysis

**Figure 5:**
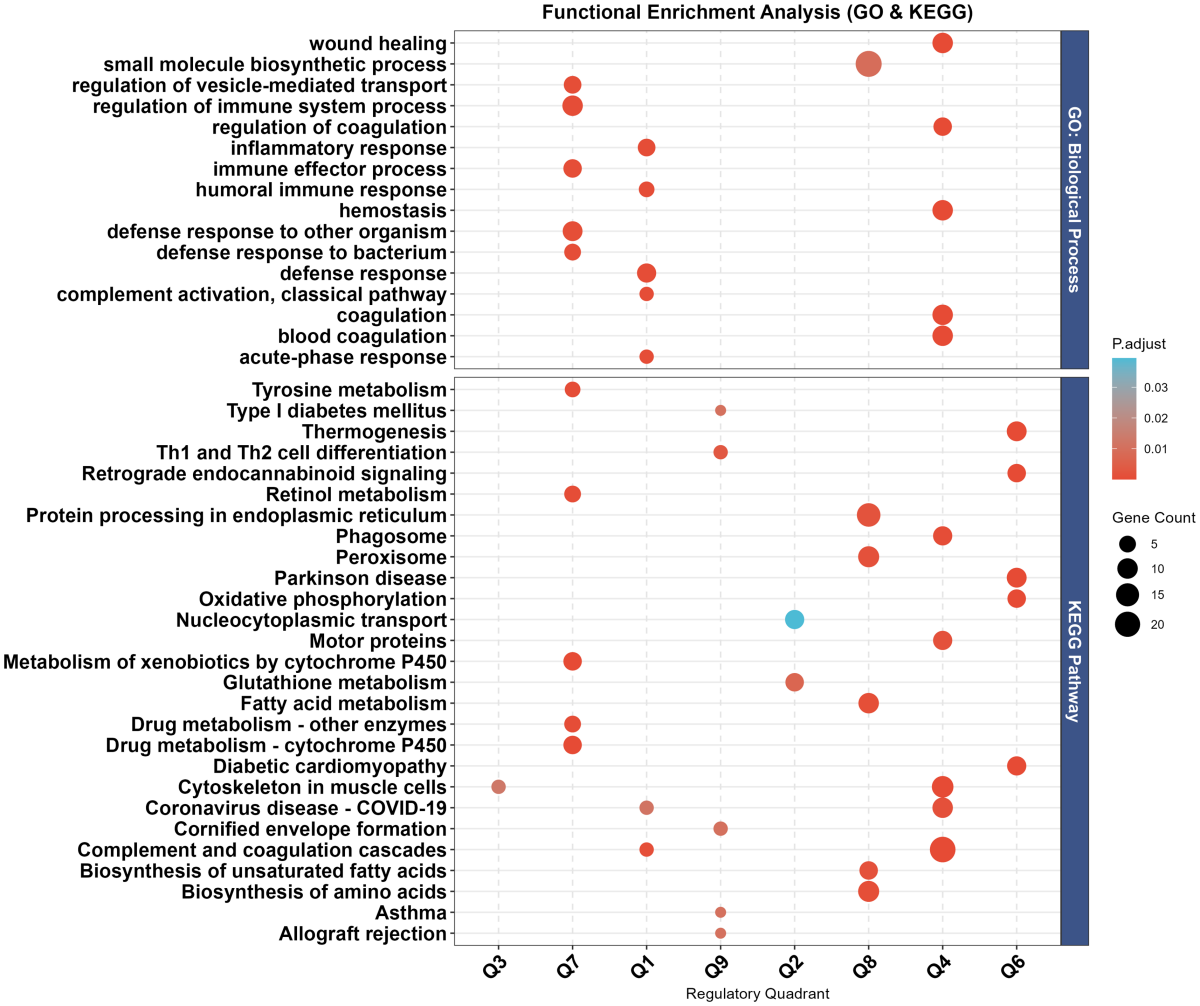
Quadrant-specific functional enrichment analysis. Dot plot of top enriched GO biological processes (top) and KEGG pathways (bottom) per quadrant. Dot size: gene count; color gradient: *Padj* (red: more significant).

Functional enrichment analysis revealed distinct profiles across quadrants (Figure 5; Supplementary Table S7).

Protein-only regulation (Q2 and Q8): Q8 (*n* = 165 genes) was enriched in Fatty acid metabolism, Biosynthesis of amino acids, Biosynthesis of unsaturated fatty acids, and Peroxisome (KEGG), as well as small molecule biosynthetic process (GO-BP). Q2 (*n* = 69 genes) was enriched in Glutathione metabolism and Nucleocytoplasmic transport.

mRNA-only regulation (Q4 and Q6): Q4 (*n* = 10 genes) was enriched in Complement and coagulation cascades, Phagosome (KEGG), blood coagulation, and wound healing (GO-BP). Q6 (*n* = 4 genes) was enriched in Thermogenesis and Oxidative phosphorylation.

Concordant regulation (Q3 and Q7): Q3 (*n* = 3 genes) was enriched in Cytoskeleton in muscle cells. Q7 (*n* = 6 genes) was enriched in Drug metabolism—cytochrome P450 (KEGG) and regulation of immune system process (GO-BP).

Discordant regulation (Q1 and Q9): Q1 (*n* = 7 genes) was enriched in humoral immune response, defense response (GO-BP), and Complement and coagulation cascades (KEGG). Q9 (*n* = 4 genes) was enriched in Th1 and Th2 cell differentiation.

### 3.8 Identification and validation of key candidate genes

#### 3.8.1 Prioritization of key candidate genes

The top 5 up-regulated and top 5 down-regulated molecules at each omics level were identified based on log₂FC magnitude (Table 5).

**Table 5:** Final key candidate genes.

Gene	Quadrant	mRNA log₂FC	Protein log₂FC	Regulatory mode
*SELENOW*	Q3	2.11	3	Concordant up
*GPX1*	Q3	0.73	1.89	Concordant up
*ULBP13*	Q4	−10.24	NS	mRNA-specific down
*F5*	Q4	−8.73	NS	mRNA-specific down
*ABLIM1*	Q2	NS	3	Protein-specific up
*SELENOH*	Q2	NS	3	Protein-specific up
*GZMB*	Q8	NS	−1.50	Protein-specific down
*ATG4B*	Q8	NS	−1.04	Protein-specific down

Top DEGs (Transcriptome): Among up-regulated DEGs, *SELENOW* showed the highest log₂FC (+2.11), followed by *SEMA3E* (+1.89), *C3H1orf146* (+1.76), *GPRC5A* (+1.53), and FGFR2 (+1.48). Among these, only *SELENOW* showed concordant protein up-regulation (protein log₂FC = +3.00). Among down-regulated DEGs, *ULBP13* showed the largest decrease (log₂FC = −10.24), followed by F5 (−8.73), *ULBP21* (−6.58), *ULBP3* (−5.92), and *CCL22* (−4.85). None of these showed significant protein-level changes.

Top DEPs (Proteome): Among up-regulated DEPs, *ABLIM1* and *SELENOH* showed the highest log₂FC (both +3.00), followed by *GPX1* (+1.89), *EEFSEC* (+1.48), and *SUOX* (+1.15). Among these, only GPX1 showed concordant mRNA up-regulation (mRNA log₂FC = +0.73). Among down-regulated DEPs, GZMB showed the largest decrease (log₂FC = −1.50), followed by *RING1* (−1.49), VPS45 (−1.22), *MAL2* (−1.20), and ATG4B (−1.04). None of these showed significant mRNA changes.

Among the 20 top-ranked molecules, 2 (10%) showed concordant regulation at both omics levels (*SELENOW* and *GPX1*), while 18 (90%) showed single-level regulation. Eight genes were selected as key candidates (Table 5).

#### 3.8.2 Cross-omics correlation of Core candidate genes

**Figure 6:**
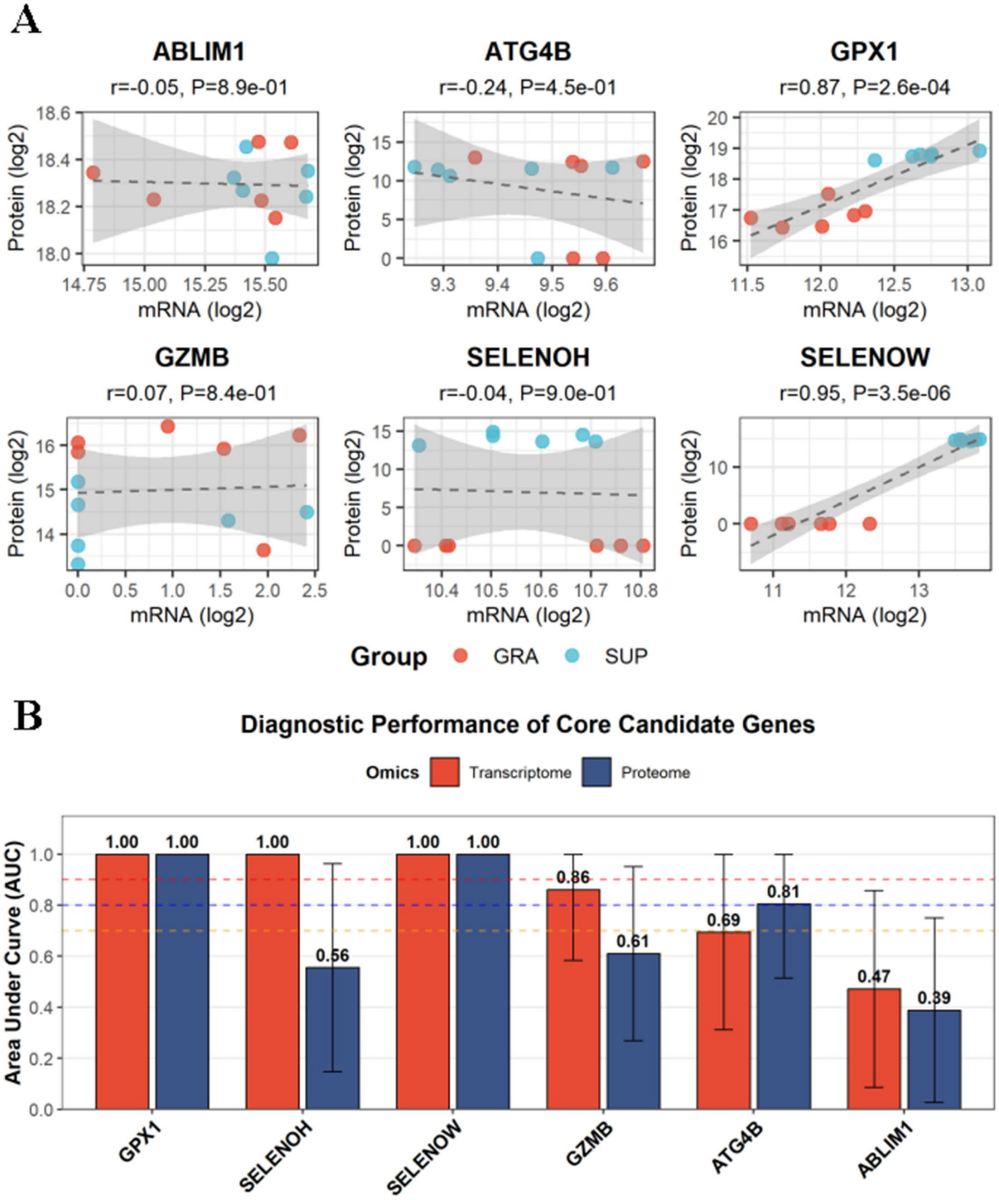
Cross-omics correlation and diagnostic performance of core candidate genes. **(A)** Scatter plots of mRNA-protein Pearson correlations for six genes. Red: GRA; blue: SUP. Dashed lines: regression fits with 95% CI. **(B)** ROC analysis showing AUC values at transcriptome (blue) and proteome (red) levels. Error bars: 95% CI. Dashed lines: AUC thresholds (0.7, 0.8, 0.9).

Pearson correlation analysis assessed the mRNA–protein expression concordance for six core candidate genes detected in both omics datasets (Figure 6A; Table 6).

**Table 6:** Cross-omics correlation and ROC diagnostic analysis results of core candidate genes.

Gene	mRNA-protein correlation *r*	*p*-value	Transcriptome AUC (95% CI)	Proteome AUC (95% CI)
*SELENOW*	0.95	3.54 × 10^−6^	1.00 (1.00–1.00)	1.00 (1.00–1.00)
*GPX1*	0.87	2.58 × 10^−4^	1.00 (1.00–1.00)	1.00 (1.00–1.00)
*GZMB*	0.07	0.84	0.61 (0.27–0.95)	0.86 (0.58–1.00)
*ABLIM1*	−0.05	0.89	0.39 (0.03–0.75)	0.47 (0.09–0.86)
*SELENOH*	−0.04	0.9	0.56 (0.15–0.96)	1.00 (1.00–1.00)
*ATG4B*	−0.24	0.45	0.81 (0.52–1.00)	0.69 (0.31–1.00)
*ULBP13*	Not detected	Not detected	1.00 (1.00–1.00)	Not detected
*F5*	Not detected	Not detected	0.83 (0.58–1.00)	Not detected

Two genes (33.3%; 2/6) showed statistically significant positive correlations (*p* < 0.05). *SELENOW* exhibited the strongest correlation (*r* = 0.95, 95% CI: 0.81–0.98, *p* = 3.54 × 10^−6^), followed by *GPX1* (*r* = 0.87, 95% CI: 0.58–0.96, *p* = 2.58 × 10^−4^). Four genes showed no significant correlations: *GZMB* (*r* = 0.07, *p* = 0.84), *ABLIM1* (*r* = −0.05, *p* = 0.89), SELENOH (*r* = −0.04, *p* = 0.90), and *ATG4B* (*r* = −0.24, *p* = 0.45).

#### 3.8.3 Diagnostic performance of core candidate genes

ROC analysis evaluated the diagnostic performance of eight core candidate genes at both omics levels (Figure 6B; Table 6).

Transcriptome level (*n* = 8 genes): The mean AUC was 0.77 ± 0.23. Three genes achieved excellent performance (AUC = 1.00): *GPX1*, *SELENOW*, and *ULBP13* (all 95% CI: 1.00–1.00). Two genes showed good performance: F5 (AUC = 0.83, 95% CI: 0.58–1.00) and ATG4B (AUC = 0.81, 95% CI: 0.52–1.00). Three genes showed poor performance: *GZMB* (AUC = 0.61, 95% CI: 0.27–0.95), *SELENOH* (AUC = 0.56, 95% CI: 0.15–0.96), and *ABLIM1* (AUC = 0.39, 95% CI: 0.03–0.75).

Proteome level (*n* = 6 genes): The mean AUC was 0.84 ± 0.22. Three genes achieved excellent performance (AUC = 1.00): *GPX1*, *SELENOH*, and *SELENOW* (all 95% CI: 1.00–1.00). One gene showed good performance: *GZMB* (AUC = 0.86, 95% CI: 0.58–1.00). Two genes showed poor performance: *ATG4B* (AUC = 0.69, 95% CI: 0.31–1.00) and *ABLIM1* (AUC = 0.47, 95% CI: 0.09–0.86).

*GPX1* and *SELENOW* achieved AUC = 1.00 at both omics levels. *SELENOH* showed AUC = 1.00 at the proteome level but AUC = 0.56 at the transcriptome level.

#### 3.8.4 Cross-omics correlation of core candidate genes

Pearson correlation analysis was performed to assess the mRNA-protein expression concordance for six core candidate genes (Figure 6A). Of the six genes analyzed, two (33.3%) showed statistically significant positive correlations (*p* < 0.05). *SELENOW* exhibited the strongest correlation (*r* = 0.95, 95% CI: 0.81–0.98, *p* = 3.54 × 10^−6^), followed by *GPX1* (*r* = 0.87, 95% CI: 0.58–0.96, *p* = 2.58 × 10^−4^). The remaining four genes showed no significant correlations: GZMB (*r* = 0.07, *p* = 0.84), ABLIM1 (*r* = −0.05, *p* = 0.89), SELENOH (*r* = −0.04, *p* = 0.90), and ATG4B (*r* = −0.24, *p* = 0.45). Three genes exhibited positive correlations and three exhibited negative correlations (Table 6).

#### 3.8.5 Diagnostic performance of core candidate genes

ROC analysis was performed to evaluate the diagnostic performance of eight core candidate genes at both transcriptome and proteome levels (Figure 6B, Table 6).

##### 3.8.5.1 Transcriptome level

The mean AUC for eight genes was 0.77. Three genes achieved excellent diagnostic performance (AUC = 1.00): *GPX1* (95% CI: 1.00–1.00), *SELENOW* (95% CI: 1.00–1.00), and *ULBP13* (95% CI: 1.00–1.00). Two genes showed good performance: *F5* (AUC = 0.83, 95% CI: 0.58–1.00) and *ATG4B* (AUC = 0.81, 95% CI: 0.52–1.00). Three genes exhibited poor diagnostic performance: *GZMB* (AUC = 0.61, 95% CI: 0.27–0.95), *SELENOH* (AUC = 0.56, 95% CI: 0.15–0.96), and *ABLIM1* (AUC = 0.39, 95% CI: 0.03–0.75).

##### 3.8.5.2 Proteome level

The mean AUC for six genes was 0.84. Three genes achieved excellent diagnostic performance (AUC = 1.00): *GPX1*, *SELENOH*, and *SELENOW* (all 95% CI: 1.00–1.00). One gene showed good performance: *GZMB* (AUC = 0.86, 95% CI: 0.58–1.00). Two genes exhibited poor diagnostic performance: ATG4B (AUC = 0.69, 95% CI: 0.31–1.00) and *ABLIM1* (AUC = 0.47, 95% CI: 0.09–0.86).

*GPX1* and *SELENOW* achieved perfect diagnostic performance (AUC = 1.00) at both omics levels. *SELENOH* showed an AUC of 1.00 at the proteome level but 0.56 at the transcriptome level. Detailed results are presented in Table 6.

## 4 Discussion

### 4.1 Coordinated activation of antioxidant defense systems

One of the most striking findings of this study was the coordinated upregulation of selenoprotein family members in the hearts of supplemented yaks. In the nine-quadrant analysis, quadrant Q3 (concordant upregulation) contained only three genes, all belonging to the selenoprotein family: *SELENOW*, *SELENOP*, and *GPX1*. Additionally, quadrant Q2 (protein-specific upregulation) was enriched with multiple selenium-related proteins, including *SELENOH*, *GPX3*, *GPX4*, *TXNRD1*, and *EEFSEC*. This coordinated upregulation pattern of multiple selenoproteins across different omics levels is relatively rare in cardiac tissue studies.

The cardioprotective roles of selenoproteins have been extensively documented. The glutathione peroxidase (*GPX*) family represents one of the most important intracellular antioxidant enzyme systems, protecting cells from oxidative damage by reducing hydrogen peroxide and lipid hydroperoxides (24–26). Previous studies have demonstrated that *GPX1* deficiency exacerbates myocardial ischemia–reperfusion injury, while *GPX1* overexpression attenuates cardiomyocyte apoptosis (27). *GPX4*, as the only *GPX* family member capable of directly reducing membrane phospholipid hydroperoxides, plays a critical role in maintaining cardiomyocyte membrane integrity (25, 28, 29). The coordinated upregulation of *GPX1*, *GPX3*, and *GPX4* at the protein level observed in this study suggests that nutritional supplementation may protect cardiomyocytes by enhancing the glutathione antioxidant system.

Notably, the upregulation of selenoproteins in this study may be directly related to improved selenium nutritional supply. Selenium is an essential trace element for selenoprotein synthesis, and its bioavailability directly affects selenoprotein translation efficiency (30–32). The Qinghai-Tibet Plateau is characterized by generally low soil selenium content, and forage selenium levels often fail to meet livestock requirements (33). During winter grazing, forage withering and reduced coverage further exacerbate the risk of inadequate selenium intake. The feed formulation used in the supplemented group likely contained higher selenium levels, thereby promoting selenoprotein synthesis. The protein-level upregulation of *EEFSEC* (selenocysteine-specific elongation factor; log₂FC = +1.48) supports this hypothesis, as this protein is a core component of the selenoprotein translation machinery (34, 35).

*SELENOW* and *GPX1* exhibited strong positive mRNA-protein correlations (*r* = 0.95 and 0.87, *p* < 0.001), whereas *SELENOH*, despite showing the largest protein upregulation (log₂FC = +3.00), displayed no significant mRNA changes. This difference suggests that selenoprotein family members may be subject to different levels of regulation: *SELENOW* and *GPX1* are primarily regulated at the transcriptional level, while *SELENOH* may depend more on translation efficiency or protein stability regulation. This observation is consistent with findings by Sunde et al. (36), who reported that different selenoproteins exhibit distinct response hierarchies to changes in selenium status, with some selenoprotein mRNA levels being relatively insensitive to selenium status while protein levels show stronger responsiveness.

From a functional perspective, the heart is an organ highly dependent on oxidative metabolism, continuously exposed to reactive oxygen species (ROS) production. High-altitude hypoxic environments further increase oxidative stress risk (37). Additionally, cold winter temperatures increase thermogenic demands, accelerating mitochondrial electron transport chain activity and consequently increasing ROS byproduct generation (38, 39). The activation of the selenoprotein system observed in this study may represent an adaptive response of cardiac tissue to enhance antioxidant defense following nutritional improvement. Proteomic functional enrichment analysis showed that upregulated proteins were significantly enriched in “Glutathione metabolism” and “Antioxidant activity” pathways, further supporting the conclusion of antioxidant system activation.

### 4.2 Predominance of post-transcriptional regulation

Another important finding of this study was the significant discordance between transcriptomic and proteomic changes. Among 4,080 matched gene-protein pairs, only 268 (6.57%) showed significant changes, of which concordant changes (Q3 + Q7) accounted for only 3.4% (9 pairs), while protein-specific changes (Q2 + Q8) reached 87.3% (234 pairs). These results indicate that post-transcriptional regulatory mechanisms play a dominant role in the cardiac response to nutritional intervention.

Incomplete correlation between mRNA and protein abundance is a universal feature of biological systems. Multiple large-scale omics studies have reported that mRNA-protein correlation coefficients typically range from 0.4 to 0.6 (40–42). However, the degree of discordance observed in this study (87.3% protein-specific changes) was markedly higher than literature-reported averages. This extreme discordance may reflect a specialized regulatory strategy of cardiac tissue under stress conditions. As a terminally differentiated organ with limited cell renewal capacity, the heart may rely more heavily on post-transcriptional mechanisms for rapid proteome adjustment rather than transcriptional reprogramming (43).

Post-transcriptional regulation can be achieved through multiple pathways. First, translation efficiency is regulated by various factors, including ribosome occupancy, mRNA secondary structure, upstream open reading frames (uORFs), and miRNA-mediated translational repression (44). Second, protein stability and degradation rates significantly impact final protein abundance. The ubiquitin-proteasome system and autophagy-lysosome pathway are the two major protein degradation pathways in eukaryotic cells (45). Interestingly, among the core candidate genes identified in this study, *ATG4B* (autophagy-related protein) showed significant protein-level downregulation (log₂FC = −1.04), which may reflect the influence of altered autophagic activity on overall proteome homeostasis.

The expression pattern of *SELENOH* provides a typical illustration of the importance of post-transcriptional regulation. This protein exhibited the largest upregulation at the proteomic level (log₂FC = +3.00) and perfect diagnostic performance (AUC = 1.00), yet its mRNA level showed no significant change, with extremely weak mRNA-protein correlation (*r* = −0.04, *p* = 0.90). At the transcriptomic level, *SELENOH* diagnostic performance was only 0.56, approaching random chance. This dramatic inter-omics difference strongly suggests that *SELENOH* abundance is primarily regulated by post-transcriptional mechanisms, potentially involving enhanced translation efficiency or reduced protein degradation. *SELENOH* has been reported to participate in redox regulation and genomic stability maintenance (46–48), and its protein-specific upregulation may represent an adaptive strategy of cardiac tissue to prioritize antioxidant protein reserves following nutritional improvement.

From a methodological perspective, the high degree of discordance observed in this study may also be partially attributable to technical factors. Transcriptomic and proteomic detection differ in dynamic range and sensitivity: RNA-seq can detect extremely low-abundance transcripts, while DIA proteomics has relatively limited detection capability for low-abundance proteins. Furthermore, the two omics datasets originated from the same tissue but not identical cell populations, and cellular heterogeneity may lead to signal dilution or bias. Nevertheless, the predominance of protein-specific changes consistently appeared across multiple independent analyses (DEPs/DEGs ratio, nine-quadrant distribution, correlation analysis), indicating that this finding has biological authenticity.

### 4.3 Adaptive adjustment of immune function

This study observed significant downregulation of multiple immune-related genes in the supplemented group, particularly *ULBP* family members. *ULBP13*, *ULBP3*, and *ULBP21* mRNA levels decreased by 10.24, 5.92, and 6.58 log₂FC units, corresponding to approximately 1,200-fold, 60-fold, and 95-fold expression differences, respectively. Such “switch-like” extreme expression changes are exceptionally rare in transcriptomic data, suggesting that these genes may serve as sensitive indicators of nutritional status.

The *ULBP* (UL16-binding protein) family comprises ligands for the *NKG2D* receptor and plays important roles in stress-induced immune surveillance (49). Under normal physiological conditions, *ULBP* family members are expressed at low levels or absent on healthy cell surfaces, but are induced upon cellular infection, DNA damage, or metabolic stress, thereby activating natural killer (*NK*) cells and cytotoxic T lymphocyte killing functions (50). The high expression of *ULBP* family members in the grazing group may reflect a cellular stress state induced by nutritional deficiency, while the significant decrease following supplementation suggests alleviation of cellular stress.

Consistent with *ULBP* family transcriptional changes, the cytotoxic effector molecule GZMB (granzyme B) also showed significant protein-level downregulation (log₂FC = −1.50). *GZMB* is the primary killing effector molecule of cytotoxic lymphocytes, eliminating damaged or abnormal cells by inducing target cell apoptosis (49, 51, 52). The decline in *GZMB* protein levels complements *ULBP* family mRNA downregulation functionally, collectively pointing toward reduced immune surveillance activity. Nine-quadrant functional enrichment analysis showed that Q4 (mRNA-specific downregulation) was enriched in “Complement and coagulation cascades,” and Q7 (concordant downregulation) was enriched in “Regulation of immune system process,” further supporting the conclusion of immune function downregulation.

However, the biological significance of immune gene downregulation requires cautious interpretation. On one hand, this may represent a beneficial adaptation whereby nutritional improvement alleviates metabolic stress, reducing unnecessary immune activation and potential autoimmune damage risk. On the other hand, decreased immune surveillance function may also weaken the body’s defense against infection or tumor cells. Given the immune-privileged status of cardiac tissue and its limited regenerative capacity, suppressing excessive immune responses may be more important for maintaining myocardial integrity (53). The cross-sectional design of this study cannot assess the long-term health effects of immune function changes, warranting further exploration in future longitudinal studies.

Coagulation factor F5 mRNA levels decreased significantly in the supplemented group (log₂FC = −8.73), second only to *ULBP13* in magnitude. *F5* encodes coagulation factor V, a key cofactor in the coagulation cascade. Interestingly, the extreme downregulation of F5 is consistent with Q4 quadrant enrichment in “Complement and coagulation cascades.” This may reflect nutritional status regulation of the hemostatic system, but specific mechanisms remain unclear and require further investigation.

### 4.4 Remodeling of lipid metabolism

Transcriptomic analysis revealed systematic downregulation of lipid metabolism-related pathways in the supplemented group. “Steroid biosynthesis” was the most significantly enriched KEGG pathway (*Padj* = 1.76 × 10–9), with all seven participating genes showing downregulation. *LDLR* (low-density lipoprotein receptor) and *SCARB1* (scavenger receptor class B member 1) were the genes with highest connectivity in the lipid metabolism network, participating in 17 and 12 enriched pathways, respectively. Additionally, key enzyme genes in the cholesterol synthesis pathway (*CYP51A1*, *DHCR7*, *MSMO1*) also showed downregulation. In proteomic analysis, Q8 (protein-specific downregulation) was enriched in “Fatty acid metabolism,” “Biosynthesis of amino acids,” and “Peroxisome” pathways, further supporting overall downregulation of metabolic activity.

The heart is an organ highly dependent on fatty acid oxidation for energy, with approximately 60–90% of ATP derived from fatty acid β-oxidation (5, 54). Downregulation of lipid metabolism-related genes appears to contradict the heart’s high energy demands. However, this change may reflect improved metabolic substrate accessibility following nutritional supplementation. Under nutrient-sufficient conditions, the body no longer needs to upregulate lipid uptake and synthesis pathways to maintain energy supply; consequently, these pathways may be downregulated to basal levels. Furthermore, lipid metabolism downregulation may be an indirect consequence of the antioxidant response, as fatty acid β-oxidation is a major source of mitochondrial ROS (55).

Notably, downregulation of *LDLR* and *SCARB1* may have complex implications for cardiovascular health. These two receptors mediate LDL and HDL uptake, respectively, and changes in their expression may affect intracellular cholesterol homeostasis. However, in healthy cardiomyocytes, cholesterol is primarily taken up via the *LDLR* pathway to maintain membrane structure and signaling molecule precursor supply (56–58). Whether the observed downregulation reflects reduced cholesterol demand or altered uptake pathways requires further elucidation through lipidomic analysis.

### 4.5 Biomarker potential of core candidate genes

Through integrative analysis, this study identified eight core candidate genes encompassing three regulatory modes: concordant upregulation (*SELENOW*, *GPX1*), mRNA-specific downregulation (*ULBP13*, *F5*), and protein-specific regulation (*ABLIM1*, *SELENOH*, *GZMB*, *ATG4B*). ROC analysis evaluated the diagnostic performance of these genes in distinguishing between supplemented and grazing groups.

*GPX1* and *SELENOW* achieved perfect diagnostic performance (AUC = 1.00) at both transcriptomic and proteomic levels, completely discriminating between the two groups. These two genes also exhibited the strongest positive mRNA-protein correlations (r = 0.87 and 0.95), indicating highly consistent expression changes across both omics levels. These characteristics make *GPX1* and *SELENOW* the most promising nutritional status biomarker candidates. From a practical application perspective, *SELENOW* showed larger transcriptional-level changes (log₂FC = +2.11 vs. +0.73 for *GPX1*), potentially offering higher detection sensitivity.

*ULBP13* also demonstrated perfect diagnostic performance at the transcriptomic level (AUC = 1.00), but was not detected in proteomic data. This may be because *ULBP13* primarily encodes a membrane protein that is difficult to adequately cover in DIA-based proteomic analysis. Nevertheless, the extreme mRNA change of *ULBP13* (log₂FC = −10.24) makes it a highly sensitive transcriptional-level marker.

The diagnostic pattern of *SELENOH* was particularly distinctive: proteomic AUC was 1.00, while transcriptomic AUC was only 0.56. This dramatic inter-omics difference again emphasizes the importance of post-transcriptional regulation while indicating that detection level should be considered when selecting biomarkers. For *SELENOH*, protein-level detection would provide far superior diagnostic value compared to mRNA detection.

It should be noted that diagnostic performance evaluation in this study was based on cardiac tissue samples, whereas clinical applications typically require non-invasive blood biomarkers. Protein products of some candidate genes (such as *GPX1*, *GPX3*) can be secreted into blood circulation and theoretically have potential for translation into blood biomarkers. However, protein abundance in blood may be influenced by multiple tissue sources, and whether tissue-specific expression patterns are reflected in blood remains to be verified. Furthermore, the limited sample size of this study (*n* = 6/group) resulted in wide confidence intervals despite some markers achieving perfect AUC (e.g., *GZMB* proteomic AUC = 0.86, 95% CI: 0.58–1.00), indicating the need for validation in larger samples.

### 4.6 Study limitations and future perspectives

Several limitations should be acknowledged. First, the limited sample size (*n* = 6 per group) constrains statistical power and may compromise biomarker robustness and generalizability. The near-perfect diagnostic performance observed in ROC analyses (AUC = 1.00 for several candidates) warrants cautious interpretation given potential overfitting risks. Second, reliance on omics data without orthogonal validation (qPCR, Western blotting, enzymatic assays) indicates that identified selenoproteins and immune molecules represent discovery-stage candidates requiring further validation. Third, the observed immune downregulation (e.g., reduced ULBP and granzyme expression) is interpreted as adaptive immunoquiescence, though alternative scenarios including compromised immune surveillance merit consideration. Fourth, the cross-sectional design and cardiac-focused approach limit assessment of temporal dynamics and systemic inter-organ responses.

Future research should prioritize expanding sample sizes with longitudinal sampling to validate candidate biomarkers, performing functional validation through *in vitro* and *in vivo* models, conducting multi-tissue comparative analyses to elucidate systemic selenoprotein networks, integrating metabolomic and lipidomic datasets for comprehensive metabolic profiling, and exploring translational potential of GPX1, SELENOW, and other candidates as minimally invasive blood biomarkers for monitoring selenium status in high-altitude livestock production.

## 5 Conclusion

Through integrative transcriptomic-proteomic analysis, this study revealed that winter nutritional supplementation remodels yak cardiac molecular regulatory networks by activating selenoprotein-mediated antioxidant systems (significant upregulation of *GPX1* and *SELENOW*) and downregulating immune surveillance molecules (*ULBP* family and *GZMB*), with post-transcriptional regulation as the predominant mechanism (87.3% protein-specific changes). *GPX1* and *SELENOW* achieved perfect diagnostic performance (AUC = 1.00) at both omics levels, qualifying as candidate molecular markers for assessing nutritional metabolic status in plateau livestock.

## Data Availability

The data presented in this study are publicly available. The data can be found here: https://ngdc.cncb.ac.cn/bioproject, accession PRJCA061310 (proteomic data) and https://ngdc.cncb.ac.cn/gsa, accession CRA040831 (transcriptomic data).
