# Supplementary figures and images for: Nutritional supplementation remodels yak cardiac molecular networks through selenoprotein activation and immune quiescence
Source: Front Nutr. 2026 Apr 14;13:1770178. doi: 10.3389/fnut.2026.1770178 (PMC13122654; doi:10.3389/fnut.2026.1770178)

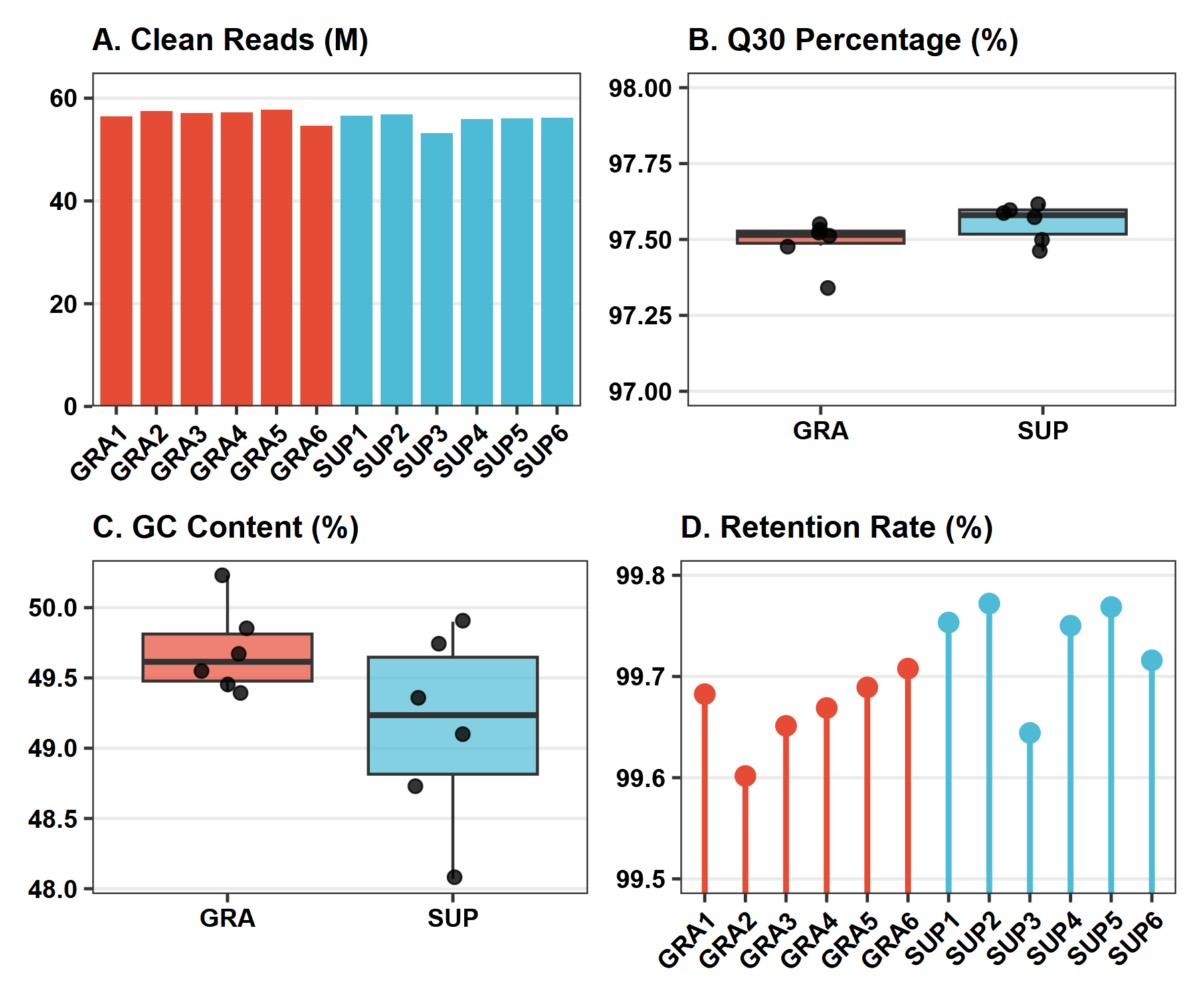

Supplement: Supplementary file 8 [file Image_1.png]

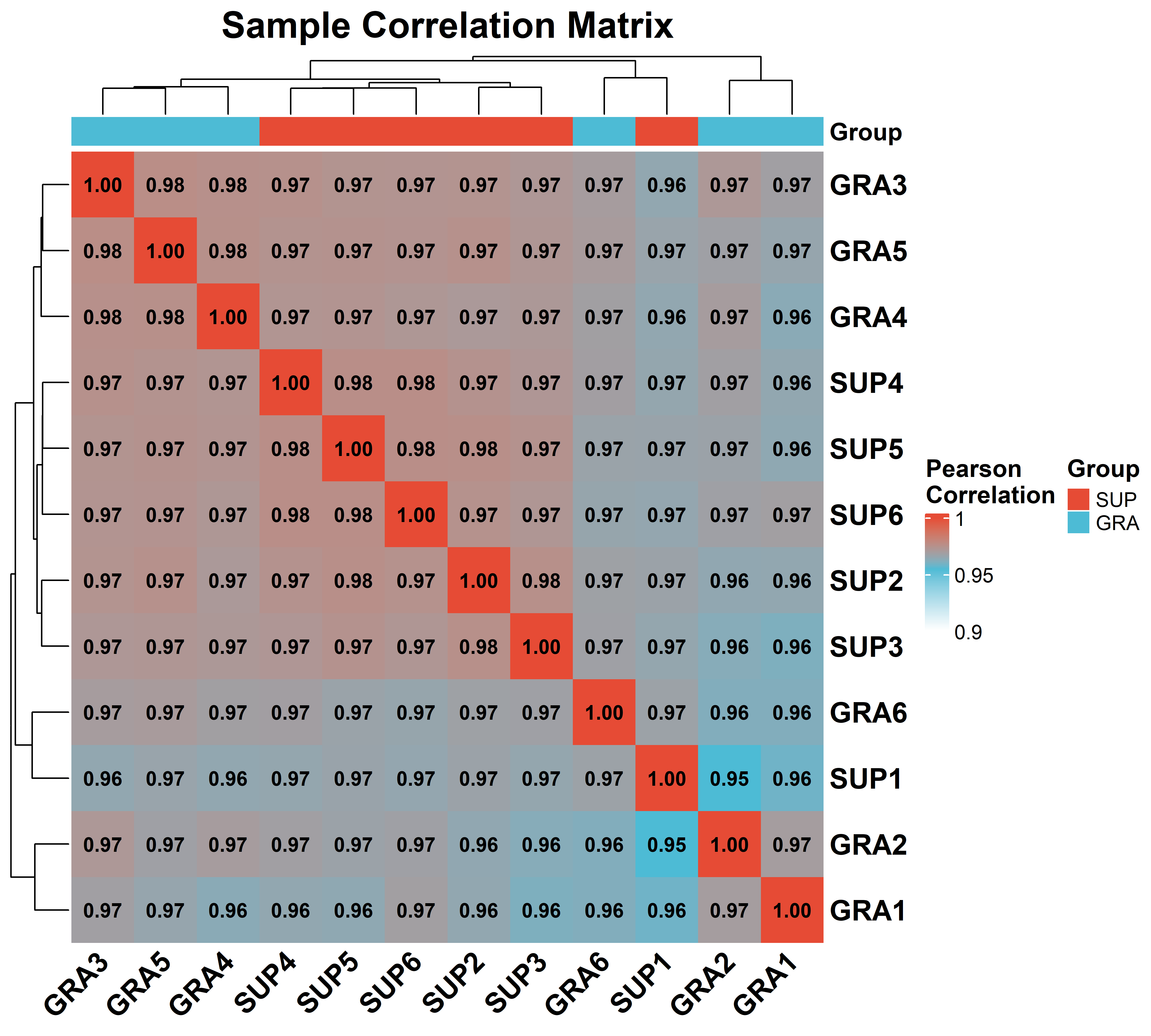

Supplement: Supplementary file 9 [file Image_2.tiff]

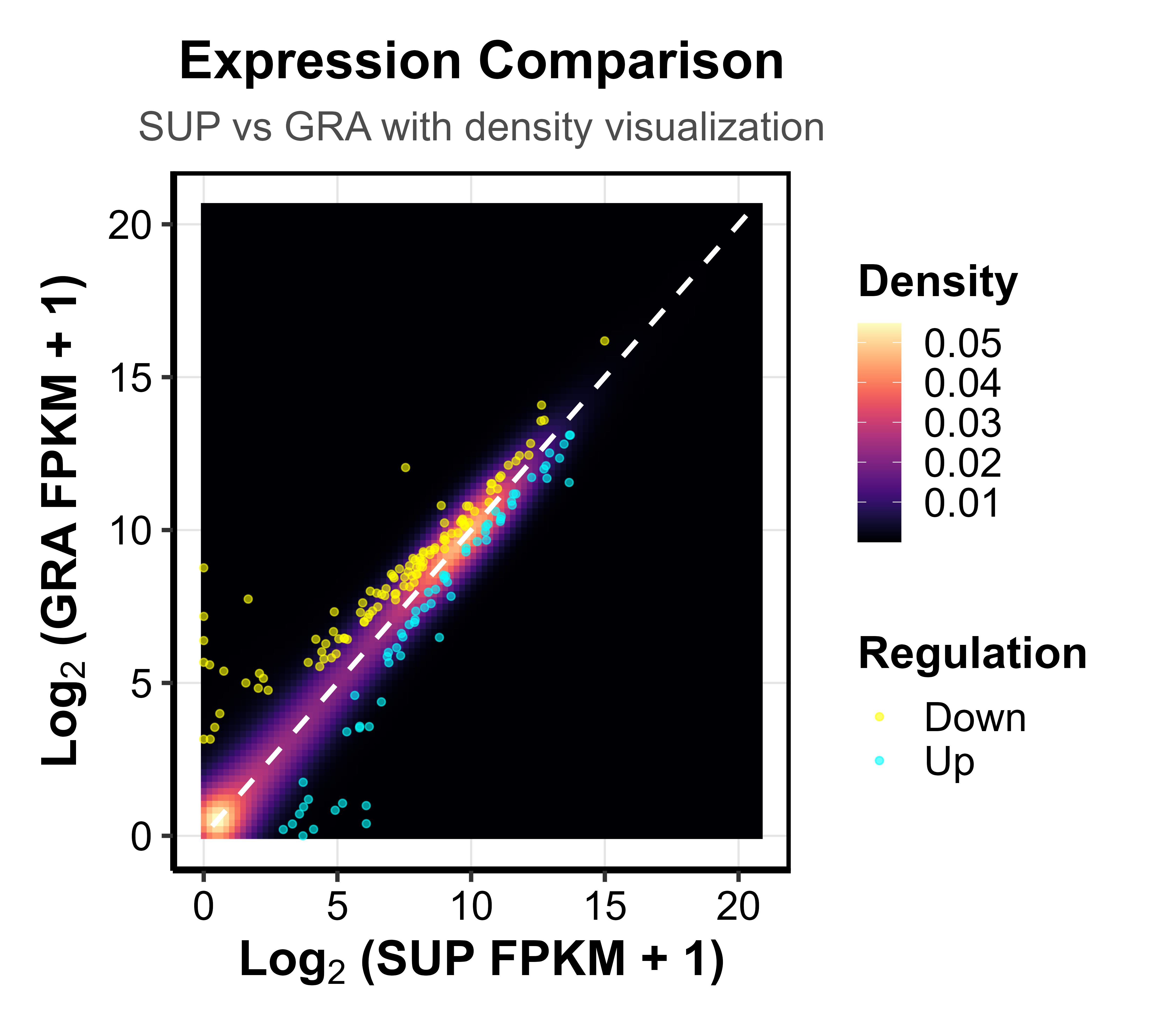

Supplement: Supplementary file 10 [file Image_3.tiff]

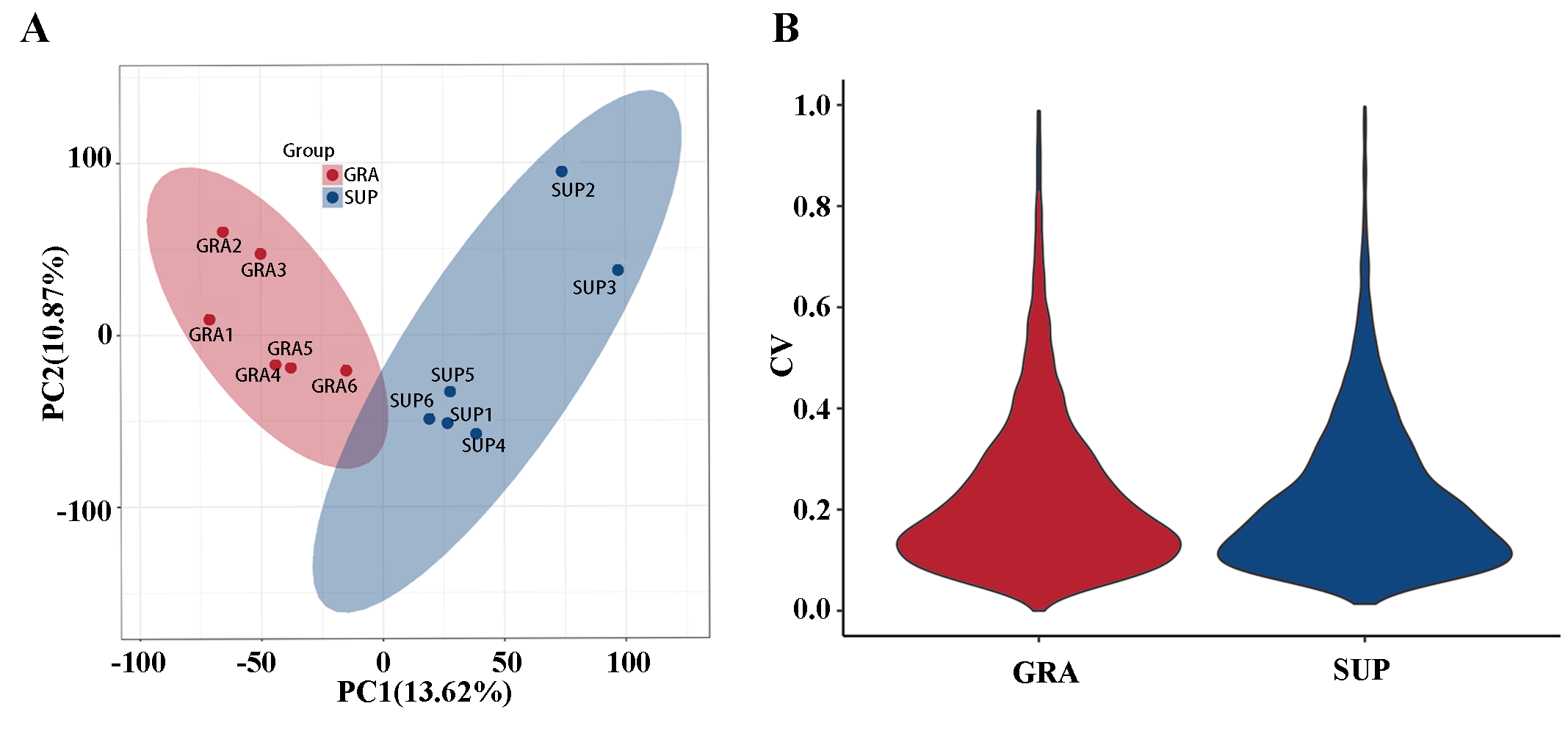

Supplement: Supplementary file 11 [file Image_4.png]
